# Supplementary material for: ‘I Knew Nothing About Parkinson’s’: Insights into Receiving a Diagnosis of Parkinson’s Disease and the Impact of Self-Management, Self-Care, and Exercise Engagement, from People with Parkinson’s and Family Members’ Perspectives: Qualitative Study
Source: Geriatrics (Basel). 2025 May 25;10(3):73. doi: 10.3390/geriatrics10030073 (PMC12192598; doi:10.3390/geriatrics10030073)
Supplement: Supplementary file 1 [file geriatrics-10-00073-s001.zip › geriatrics-3584255-supplementary.pdf]

Supplementary Material

S1: COREQ: Consolidated criteria for reporting qualitative research: a 32-item checklist for interviews and focus groups

**Section/Topic**	**Item No**	**Checklist item**	**Reported on page No**
**Domain 1: Research team and reflexivity**			
Personal Characteristics			
Interviewer/facilitator	1	Which author/s conducted the interview or focus group?Interviewer/facilitator	Pg 4, line 142
Credentials	2	What were the researcher's credentials? E.g. PhD, MD	Pg 4, line 142
Occupation	3	What was their occupation at the time of the study?	Pg 4, line 143
Gender	4	Was the researcher male or female?	Not reported
Experience and training	5	What experience or training did the researcher have? Relationship with participants	Pg 4, line 143
Relationship with participants			
Relationship established	6	Was a relationship established prior to study commencement?	Pg 5 line 227-228
Participant knowledge of the interviewer	7	What did the participants know about the researcher? e.g. personal goals, reasons for doing the research	Pg 13 line 612-617
Interviewer characteristics	8	What characteristics were reported about the interviewer/facilitator? e.g. Bias, assumptions, reasons and interests in the research topic	Pg 13 line 615-617
**Domain 2: study design**			
Theoretical framework			
Methodological orientation and Theory	9	What methodological orientation was stated to underpin the study? e.g. grounded theory, discourse analysis, ethnography, phenomenology, content analysis	Pg 4 line 179; pg 5 line 189-193
Participant selection			
Sampling	10	How were participants selected? e.g. purposive, convenience, consecutive, snowball	Pg 3 line 122-123
Method of approach	11	How were participants approached? e.g. face-to-face, telephone, mail, email	Pg 3 line 128-131
Sample size	12	How many participants were in the study?	Pg 5 line 214-216
Non-participation	13	How many people refused to participate or dropped out? Reasons?	Pg 5 line 214-216
Setting of data collection	14	Where was the data collected? e.g. home, clinic, workplace	Pg 4 line 143-144
Presence of non-participants	15	Was anyone else present besides the participants and researchers?	Not reported
Description of sample	16	What are the important characteristics of the sample? e.g. demographic data, date	Pg 6 Table 1

Data collection			
Interview guide	17	Were questions, prompts, guides provided by the authors? Was it pilot tested?	Supplementary data
Repeat interviews	18	Were repeat interviews carried out? If yes, how many?	N/A
Audio/visual recording	19	Did the research use audio or visual recording to collect the data?	Pg 4 line 147-149
Field notes	20	Were field notes made during and/or after the interview or focus group?	Pg 4 line 147
Duration	21	What was the duration of the interviews or focus group?	Pg 4 144-145
Data saturation	22	Was data saturation discussed?	Pg 4 line 151-153
Transcripts returned	23	Were transcripts returned to participants for comment and/or correction?	Pg 4 line 148-150
Domain 3: analysis and findingsz<br>Data analysis			
Number of data coders	24	How many data coders coded the data?	Pg 5 line 194-204
Description of the coding tree	25	Did authors provide a description of the coding tree?	Pg 6 Figure 1
Derivation of themes	26	Were themes identified in advance or derived from the data?	Pg 5 line 190-194
Software	27	What software, if applicable, was used to manage the data?	Pg 5 line 190
Participant checking	28	Did participants provide feedback on the findings?	Pg 4 line 148-150
Reporting			
Quotations presented	29	Were participant quotations presented to illustrate the themes / findings? Was each quotation identified? e.g. participant number	Pg 8-13; Pg 13 Table 2
Data and findings consistent	30	Was there consistency between the data presented and the findings?	Yes, Pg 8-13
Clarity of major themes	31	Were major themes clearly presented in the findings?	Yes, Pg 6 Figure 1; Pg 13 Table 2
Clarity of minor themes	32	Is there a description of diverse cases or discussion of minor themes?	Yes Pg 8-13

S2: Interview Questions: Semi-structured interviews: People with Parkinson's disease

The topic guide for these interviews will be mapped to the 14 domains of the Theoretical Domains Framework (TDF)

Overview

Confirm consent to conduct and record the interview

Introductions and expression of gratitude to the participants

Reiterating the aims of the study

Encourage individuals to be open and honest about their experiences and give as much information as possible

Participant to state name, age, years since diagnosis (first symptoms), and whether they are independent with ADLs and living with someone or alone.

Knowledge, Skills, and Memory, attention, and decision processes (TDF Domains 1, 2 and 10)

1. When you were told you had Parkinson's disease/received your diagnosis did you receive adequate information regarding your diagnosis of Parkinson's disease? How did this effect/influence you/your ability to manage your condition?

1a. Were you surprised? Can you tell me about that?

2. Has anyone talked to you about exercise and physical activity? Can you tell me about that?

Probing Question: Do you think exercise and physical activity is important for you? Why?

Probe their understanding of why exercise is important – is it more on a well-being point of view? Or a neuroprotective point of view.

3. What do you think are the most important things for you when managing your condition?

4. Have you been informed of the role of exercise in the management of Parkinson's disease? If so, by who? How did this impact your outlook?

5. Is exercise important to you? Can you explain why/why not?

6. Do you believe you benefit from exercise? Can you explain?

7. Are you aware of the World Health Organisation's (or any) guidelines on physical Activity? Can you explain this

Belief about capabilities, Optimism, and Belief about consequences (TDF Domains 4, 5 and 6)

8. What activities do you consider to be "exercise"?

9. Do you engage in exercise?

- If no, can you please explain why

- If yes, how many times a week to you exercise? What type of exercise do you enjoy?

10. Do you prefer to exercise alone? With a family member/friend? As part of a group?

11. Other than physically, do you believe you benefit from exercise/how does exercise benefit you? Can you explain?

Symptom Management

12. Can you discuss some Parkinson's disease symptoms (both motor and non-motor) you have experienced? How do you manage these symptoms?

13. Do your symptoms effect your participation in everyday life? Can you explain?

14. How does exercise effect your Parkinson's disease symptoms?

15. How do your symptoms effect your ability to exercise?

Reinforcement, Emotion and Behaviour regulation (TDF Domains 7, 13 and 14)

16. What motivates you to exercise? /What encourages you to engage in exercise?

17. What reduces your motivation?

18. How do you stay motivated (strategies)? What techniques do you use to stay motivated?

19. As healthcare professionals what can we offer you or others to help you stay motivated/improve your motivation?

Barriers and Facilitators

20. Do you find it hard to exercise? If so, why?

Probe both personal and environment factors

Probe both motor and non-motor

Follow up question: How do you overcome this?

21. Do you believe it is easy for you to engage in exercise services in the community? Can you explain?

22. How can we make exercise services more accessible for you?

Intentions and Goals (TDF Domains 8 and 9)

23. Why do you exercise?

Probe the benefits, social element

24. Do you set goals for yourself? Can you give an example?

25. Are you as physically active as you would like to be?

If no, why not? What can you do to change this?

Environmental context and resources, social influences and social role and Identity (TDF Domains 11, 12 and 3)

26. Do you feel supported by your family and friends? Can you explain?

27. Do you feel supported within your community? Can you explain?

28. Do you believe the people you encounter during your everyday life understand your Parkinson's disease specific needs? Can you explain/How does this effect you?

29. What are the general society beliefs regards Parkinson's disease? How does this effect you?

30. Do you belief society facilitates/provides for your Parkinson's disease specific needs?

31. How can we improve this/What can we do to improve this?
Conclude

32. Ask participant if there is anything else they would like to say/add

33. Thank them for their participation.

S3: Interview Questions; Interview Group: Family Member/Carers

The topic guide for this interview will be mapped to the 14 domains of the Theoretical Domains Framework (TDF)

Overview

Confirm consent to conduct and record the focus group

Introductions and expression of gratitude to the participants

Reiterating the aims of the study

Encourage individuals to be open and honest about their experiences and give as much information as possible

Participant to state name, their family-member/participant's name, if they are a family member or carer and how long ago since their family member was diagnosed

Introduction

1. What are your experiences of living/caring for someone with Parkinson's disease?

2. Does your family member engage in exercise? Can you explain (type, duration, frequency)?

3. Do you believe your family member benefits from/enjoys exercise? Can you explain?

4. How important is exercise to your family member?

5. What is your role in helping your family member to exercise/ Do you play a role in helping your family member to exercise?

6. What are the strengths and weaknesses to your role in helping your family member to exercise?

Knowledge and Skills (TDF Domains 1 and 2)

7. When your family member received their diagnosis did you receive adequate information and education regarding the management of Parkinson's disease?

8. Were you educated regarding the role of exercise in the management of

Parkinson's disease? If so, by who? How did this influence your outlook?

9. Is exercise important to your family member? Can you explain why/why not?

10. Do you believe your family member benefits from exercise? Can you explain?

11. What are your thoughts and understanding of the role of exercise in the management of Parkinson's disease?

12. As healthcare professionals, what can we do to help?

13. Are you aware of the World Health Organisation's guidelines on physical Activity? Can you explain this?

Reinforcement, Emotion and Behaviour Regulation (TDF Domains 7, 13 and 14)

14. What challenges does Parkinson's disease bring to exercise? How do you help overcome these?

Probe personal and environment factors; motor and non-motor

15. What are the barriers and possible solutions to helping your family member to exercise? How can we help?

16. As healthcare professionals how can we help overcome these challenges?

17. Can you discuss some examples of motivational technique you find beneficial to use to encourage exercise?

Intentions and Goals (TDF Domains 8 and 9)

18. Does your family member set personal goal when it comes to exercise? Can you explain?

19. What role do you play in the goal setting process? Can you explain?

20. Do you think your family member is as physically active as they would like to be? If no, why not? What can you do to help them change this?

Environmental Context and resources, social influences and social role and Identity (TDF Domains 11, 12 and 3)

21. Do you believe it is easy for your family member to access exercise services in the community? Can you explain?

22. Do you feel your family member is supported within your community? Can you explain?

23. What are the general society beliefs regarding Parkinson's disease? How does this influence your family- member?

24. Do you belief society facilitates for people with Parkinson's disease?

25. How can we improve this?

Conclude

26. Ask participants if there is anything else they would like to say/add

27. Thank them for their participation.
